# Intensive care unit versus high-dependency care unit for patients with acute heart failure: a nationwide propensity score-matched cohort study

**DOI:** 10.1186/s40560-021-00592-2

**Published:** 2021-12-20

**Authors:** Hiroyuki Ohbe, Hiroki Matsui, Hideo Yasunaga

**Affiliations:** Department of Clinical Epidemiology and Health Economics, School of Public Health, The University of Tokyo, 7-3-1 Hongo, Bunkyo-ku, Tokyo, 1130033 Japan

**Keywords:** Cardiac intensive care, Heart failure, Intensive care unit, High-dependency care unit, Propensity score, Administrative database

## Abstract

**Background:**

A structure and staffing model similar to that in general intensive care unit (ICUs) is applied to cardiac intensive care unit (CICUs) for patients with acute heart failure. However, there is limited evidence on the structure and staffing model of CICUs. The present study aimed to assess whether critical care for patients with acute heart failure in the ICUs is associated with improved outcomes than care in the high-dependency care units (HDUs), the hospital units in which patient care levels and costs are between the levels found in the ICU and general ward.

**Methods:**

This nationwide, propensity score-matched, retrospective cohort study was performed using a national administrative inpatient database in Japan. We identified all patients who were hospitalized for acute heart failure and admitted to the ICU or HDU on the day of hospital admission from April 2014 to March 2019. Propensity score-matching analysis was performed to compare the in-hospital mortality between acute heart failure patients treated in the ICU and HDU on the day of hospital admission.

**Results:**

Of 202,866 eligible patients, 78,646 (39%) and 124,220 (61%) were admitted to the ICU and HDU, respectively, on the day of admission. After propensity score matching, there was no statistically significant difference in in-hospital mortality between patients who were admitted to the ICU and HDU on the day of admission (10.7% vs. 11.4%; difference, − 0.6%; 95% confidence interval, − 1.5% to 0.2%). In the subgroup analyses, there was a statistically significant difference in in-hospital mortality between the ICU and HDU groups among patients receiving noninvasive ventilation (9.4% vs. 10.5%; difference, − 1.0%; 95% confidence interval, − 1.9% to − 0.1%) and patients receiving intubation (32.5% vs. 40.6%; difference, − 8.0%; 95% confidence interval, − 14.5% to − 1.5%). There were no statistically significant differences in other subgroup analyses.

**Conclusions:**

Critical care in ICUs was not associated with lower in-hospital mortality than critical care in HDUs among patients with acute heart failure. However, critical care in ICUs was associated with lower in-hospital mortality than critical care in HDUs among patients receiving noninvasive ventilation and intubation.

**Supplementary Information:**

The online version contains supplementary material available at 10.1186/s40560-021-00592-2.

## Background

The numbers of critically ill patients with acute heart failure in cardiac intensive care units (CICU) have been dramatically increasing during the past few decades [1–3]. Management of acute heart failure using advanced drugs (e.g., intravenous vasodilators, inotropes, and vasopressors) and invasive organ supportive therapies (mechanical ventilation, mechanical circulatory support, and renal replacement therapy) has become a major focus of modern CICUs [4].

Based on the evidence gathered from general intensive care units (ICUs) [5–7], several academic societies have recommended a structure and staffing model for CICUs similar to that of general ICUs; e.g., intensivist staffing, a closed ICU model, and a nurse-to-patient ratio of 1:1 to 1:2 [4, 8–10]. However, evidence for these recommendations is limited when focusing on the structure and staffing model of CICUs [11, 12]. No clinical trials and only one observational study has shown that care by cardiac intensivists, compared with care by senior residents in internal medicine, is associated with reduced mortality in CICUs [13]. The benefit of critical care may vary depending on the severity of critical illness [7, 14], but no study has examined effect modification in the subset of patients with acute heart failure admitted to the CICU.

ICUs are the hospital units that provide the most advanced critical care, whereas high-dependency care units (HDUs) are the hospital units in which patient care levels and costs are between the levels found in the ICU and general ward [7]. CICUs are the hospital units that provide critical care for patients with cardiovascular diseases using various structure and staffing models, and many Japanese hospitals currently use some beds in their ICUs and HDUs as CICU beds for patients with acute heart failure because of the lack of insurance reimbursement criteria that specify what qualifies as a CICU [8, 9, 15]. Therefore, the current situation in Japan can provide a unique opportunity to assess the structure and staffing model of CICUs. Using a national inpatient database in Japan, we assessed the survival benefit of patients with acute heart failure who were admitted to the ICU versus HDU.

## Methods

### Data source

This was a nationwide, propensity score-matched, retrospective cohort study using the Diagnosis Procedure Combination database, a national inpatient database in Japan. The Institutional Review Board of The University of Tokyo approved this study (approval number, 3501-3; December 25, 2017).

The database contains discharge abstracts and administrative claims data from voluntarily participating hospitals [16]. In 2017, the database contained data for about 75% of all ICU beds and 70% of all HDU beds in Japan [15]. This database includes the following patient-level data for all hospitalizations: demographic characteristics; diagnoses recoded with International Classification of Diseases, Tenth Revision (ICD-10) codes; daily procedures recorded using Japanese medical procedure codes; daily drug administrations; and admission and discharge status. A previous validation study for this database showed high specificity and moderate sensitivity for recorded diagnoses and high specificity and sensitivity for recorded procedures [17].

We also used facility information and statistics data from the Survey of Medical Institutions 2017 [18]. We combined these data with the Japanese Diagnosis Procedure Combination inpatient database using a specific hospital identifier. The Survey of Medical Institutions included the type of ward (e.g., general, ICU, or HDU), number of hospital beds in each ward, and hospital type (i.e., tertiary emergency hospital or academic hospital).

### Study population

We identified all patients who were hospitalized for acute heart failure (ICD-10 codes I099, I110, I130, I132, I255, I420, I425-I429, I43x, or I50x) and were admitted to the ICU or HDU from April 1, 2014, to March 31, 2019. The sensitivity and specificity of the diagnosis of acute heart failure in the database was 68.8% and 97.5%, respectively [17]. We excluded patients (i) aged < 15 years, (ii) who were not admitted to the ICU or HDU on the day of hospital admission, and (iii) who were admitted to hospitals that could not be combined with data from the Survey of Medical Institutions 2017. All patients were followed up until they died or were discharged from the hospital.

### Treatment groups

Patients who were admitted to the ICU on the day of hospital admission were defined as the ICU group. Patients who were admitted to the HDU on the day of hospital admission were defined as the HDU group. We compared the patients in the ICU group with those in the HDU group. The definition of ICU in this study was a separate unit providing critical care services with at least one physician on site 24 h per day, at least two intensivists working full-time (required only for resource-rich ICUs), around-the-clock nursing, the equipment necessary to care for critically ill patients, and a nurse-to-patient ratio of 1:2. An HDU, also called an “intermediate care unit” or “step-down unit”, is area where patient care levels and costs are between the levels found in the ICU and in the general ward [7, 19, 20]. The definition of HDU in this study was almost the same as that of ICU, but an HDU had a nurse-to-patient ratio of 1:4 or 1:5 and no requirement for intensivist staffing. We present the Japanese procedure codes used to define ICUs and HDUs in Additional file 1: Table S1.

### Outcomes

The primary outcome was in-hospital mortality. The secondary outcomes were the length of hospital stay; length of ICU/HDU stay; total hospitalization costs (with 1 United States dollar equivalent to 110 Japanese yen); and complications including pneumonia, stroke, endoscopic hemostasis for gastrointestinal bleeding, catheter-related bloodstream infection, and *Clostridioides difficile* infection.

### Covariates

The covariates were age, sex, smoking history, body mass index at admission, Japan Coma Scale score at admission [21], physical function measured by the Barthel index score at admission [22], cognitive function before admission, home medical care before admission, location before admission, ambulance use, admission on a weekend (i.e., on Saturday or Sunday), comorbidities, Charlson comorbidity index score, treatments on the day of admission, and hospital characteristics.

### Statistical analysis

We performed a propensity score-matching analysis to compare the outcomes between the ICU and HDU groups [23]. A multivariable logistic regression model using all the covariates listed in Table 1 was employed to compute the propensity scores for patients who were admitted to the ICU on the day of hospital admission. One-to-one nearest-neighbor matching without replacement was then performed for the estimated propensity scores using a caliper width set at 20% of the standard deviation of the propensity scores [23]. To assess the performance of the matching, the covariates were compared using standardized differences, with absolute standardized differences of ≤ 10% considered to denote negligible imbalances between the two groups [24]. After the propensity score matching, the primary and secondary outcomes for the two groups were assessed through a generalized linear model accompanied by cluster-robust standard errors with hospitals as the clusters. Differences and their 95% confidence intervals were calculated with generalized linear models using the identity link function, irrespective of outcome types.

**Table 1:** Baseline characteristics before and after propensity score matching

Characteristics	Before propensity score matching			After propensity score matching		
	ICU	HDU	ASD	ICU	HDU	ASD
	(*n* = 78,646)	(*n* = 124,220)		(*n* = 62,352)	(*n* = 62,352)	
Age category, years						
15–59	8623 (11)	9551 (8)	11	6012 (10)	6233 (10)	1
60–69	12,549 (16)	15,166 (12)	11	9097 (15)	9182 (15)	0
70–79	21,590 (27)	29,166 (23)	9	16,293 (26)	16,534 (27)	1
80–89	27,818 (35)	49,134 (40)	9	23,419 (38)	23,159 (37)	1
≥ 90	8066 (10)	21,203 (17)	20	7531 (12)	7244 (12)	1
Male	45,539 (58)	65,536 (53)	10	34,891 (56)	35,036 (56)	1
Smoking history						
Nonsmoker	39,997 (51)	69,204 (56)	10	32,837 (53)	32,462 (52)	1
Current/past smoker	28,244 (36)	39,154 (32)	9	21,569 (35)	21,841 (35)	1
Unknown	10,405 (13)	15,862 (13)	1	7946 (13)	8049 (13)	1
Body mass index at admission, kg/m^2^						
< 18.5	11,398 (14)	20,731 (17)	6	9487 (15)	9462 (15)	0
18.5–24.9	42,295 (54)	66,216 (53)	1	33,419 (54)	33,515 (54)	0
25.0–29.9	13,836 (18)	20,142 (16)	4	10,615 (17)	10,595 (17)	0
≥ 30.0	4786 (6)	6627 (5)	3	3653 (6)	3666 (6)	0
Missing	6331 (8)	10,504 (8)	2	5178 (8)	5114 (8)	0
Japan Coma Scale score at admission						
Alert	55,363 (70)	90,007 (72)	5	45,087 (72)	45,263 (73)	1
Confusion	14,730 (19)	25,663 (21)	5	11,892 (19)	11,696 (19)	1
Somnolence	3886 (5)	4368 (4)	7	2638 (4)	2645 (4)	0
Coma	4667 (6)	4182 (3)	12	2735 (4)	2748 (4)	0
Physical function at admission						
Total/severe dependence (BI 0–60)	44,788 (57)	75,717 (61)	8	36,189 (58)	35,992 (58)	1
Slight/moderate dependence (BI 61–99)	2886 (4)	7479 (6)	11	2652 (4)	2515 (4)	1
Independent (BI 100)	14,395 (18)	18,982 (15)	8	10,864 (17)	11,160 (18)	1
Missing	16,577 (21)	22,042 (18)	8	12,647 (20)	12,685 (20)	0
Cognitive function before admission						
No dementia	62,514 (79)	87,997 (71)	20	48,040 (77)	48,328 (78)	1
Mild dementia	11,281 (14)	23,981 (19)	13	9907 (16)	9729 (16)	1
Moderate/severe dementia	4,851 (6)	12,242 (10)	14	4405 (7)	4295 (7)	1
Home medical care before admission	4628 (6)	9537 (8)	7	4030 (6)	4044 (6)	0
Location before hospitalization						
Home	69,582 (88)	108,333 (87)	4	55,212 (89)	55,371 (89)	1
Other hospitals	5069 (6)	5481 (4)	9	3430 (6)	3418 (5)	0
Nursing home	3995 (5)	10,406 (8)	13	3710 (6)	3563 (6)	1
Ambulance use	57,243 (73)	76,205 (61)	25	43,223 (69)	43,657 (70)	2
Admission on a weekend	19,860 (25)	28,864 (23)	5	15,380 (25)	15,337 (25)	0
Comorbidities						
Ischemic heart disease	23,479 (30)	32,383 (26)	8	17,885 (29)	18,039 (29)	1
Diabetes mellitus	25,442 (32)	35,323 (28)	9	19,500 (31)	19,644 (32)	1
Hypertension	42,660 (54)	64,774 (52)	4	33,757 (54)	33,743 (54)	0
Hyperlipidemia	19,133 (24)	26,527 (21)	7	14,714 (24)	14,788 (24)	0
Atrial flutter/fibrillation	18,157 (23)	35,068 (28)	12	15,533 (25)	15,433 (25)	0
Chronic kidney disease	13,750 (17)	19,434 (16)	5	10,384 (17)	10,327 (17)	0
Cancer	3429 (4)	5646 (5)	1	2796 (4)	2767 (4)	0
Charlson comorbidity index	1.4 ± 1.4	1.3 ± 1.4	7	1.4 ± 1.3	1.4 ± 1.4	0
Treatments on day of admission						
Respiratory support						
No supplemental oxygen	13,848 (18)	21,594 (17)	1	12,005 (19)	12,106 (19)	0
Supplemental oxygen	24,938 (32)	71,027 (57)	53	24,121 (39)	23,535 (38)	2
Noninvasive ventilation	31,769 (40)	28,080 (23)	39	23,015 (37)	23,351 (37)	1
Intubation	8091 (10)	3519 (3)	31	3211 (5)	3360 (5)	1
Intravenous vasodilator						
Carperitide	28,527 (36)	41,402 (33)	6	22,282 (36)	22,343 (36)	0
Nitrate	33,780 (43)	35,205 (28)	31	23,851 (38)	24,297 (39)	2
Nicorandil	5742 (7)	4894 (4)	15	3533 (6)	3598 (6)	1
Calcium-channel blocker	9730 (12)	11,468 (9)	10	6980 (11)	7018 (11)	0
Diuretic						
Intravenous furosemide	52,132 (66)	84,840 (68)	4	42,043 (67)	41,961 (67)	0
Tolvaptan	5980 (8)	11,935 (10)	7	5152 (8)	4945 (8)	1
Inotrope						
Milrinone	1059 (1)	1206 (1)	4	687 (1)	687 (1)	0
Pimobendan	1473 (2)	2332 (2)	0	1203 (2)	1216 (2)	0
Dobutamine	11,582 (15)	12,769 (10)	14	7580 (12)	7706 (12)	1
Vasopressor						
Dopamine	5254 (7)	5419 (4)	10	3205 (5)	3198 (5)	0
Noradrenaline	5871 (7)	2782 (2)	25	2360 (4)	2399 (4)	0
Mechanical circulatory support						
Intra-aortic balloon pumping	1818 (2)	538 (0)	16	502 (1)	519 (1)	0
Extracorporeal membrane oxygenation	375 (0)	101 (0)	8	88 (0)	97 (0)	0
Other treatment						
Coronary angiography	4252 (5)	2503 (2)	18	2116 (3)	2115 (3)	0
Percutaneous coronary intervention	1317 (2)	601 (0)	12	539 (1)	545 (1)	0
Digoxin	3128 (4)	4979 (4)	0	2535 (4)	2596 (4)	1
Intravenous beta-blockers	3257 (4)	4056 (3)	5	2336 (4)	2365 (4)	0
Amiodarone	3640 (5)	3712 (3)	9	2369 (4)	2431 (4)	1
Cardiac pacing	1019 (1)	814 (1)	7	599 (1)	603 (1)	0
Intermittent renal replacement therapy	3423 (4)	3370 (3)	9	2384 (4)	2430 (4)	0
Continuous renal replacement therapy	2385 (3)	684 (1)	19	672 (1)	663 (1)	0
Antibiotics	18,597 (24)	24,182 (19)	10	13,037 (21)	13,067 (21)	0
Morphine	2421 (3)	2015 (2)	10	1530 (2)	1597 (3)	1
Red blood cell transfusion	3352 (4)	3660 (3)	7	2108 (3)	2090 (3)	0
Hospital characteristics						
Tertiary emergency hospital	41,117 (52)	83,724 (67)	31	35,228 (56)	34,513 (55)	2
Academic hospital	13,678 (17)	11,775 (9)	23	8643 (14)	8985 (14)	2
Hospital volume, patients per year						
Low (≤ 66)	28,467 (36)	38,650 (31)	11	22,141 (36)	22,355 (36)	1
Medium (67–129)	28,609 (36)	39,238 (32)	10	21,325 (34)	21,692 (35)	1
High (≥ 130)	21,570 (27)	46,332 (37)	21	18,886 (30)	18,305 (29)	2

Data are presented as n (%) or mean ± standard deviation

*ICU* intensive care unit; *HDU* high-dependency care unit; *ASD* absolute standardized mean difference; *BI* Barthel index

### Subgroup analyses

We were interested in identifying the subsets of patients who would benefit most from a higher level of critical care. Therefore, based on previous studies [7, 14], we tested the potential for effect modification of ICU admission on in-hospital mortality according to treatment (respiratory support, intravenous vasodilators, diuretics, inotropes, vasopressors, renal replacement therapy, and mechanical circulatory support) on the day of admission. We performed these subgroup analyses among the propensity score-matched cohort created in the main analysis.

### Sensitivity analyses

We performed two sensitivity analyses. First, the decision about which unit to which the patient would be assigned was made by the individual physician, with no specific criteria for one or the other, leading to likely confounding by indication. Therefore, we performed sensitivity analyses to compare primary and secondary outcomes excluding patients admitted to hospitals with both ICU beds and HDU beds. In these sensitivity analyses, the attending physicians had no choice of whether to admit patients to an ICU bed or HDU bed.

Second, there are two types of ICUs in Japan: resource-rich ICUs, which have two or more intensivists working as full-time employees, ≥ 20 m^2^ per ICU bed, and a medical engineer in the hospital 24 h per day; and other standard ICUs. The structure and staffing model in resource-rich ICUs are different from those in standard ICUs, especially in terms of the intensivist staffing requirement. Therefore, we performed sensitivity analyses to compare primary outcome between patients in resource-rich ICUs versus HDUs, standard ICUs versus HDUs, and resource-rich ICUs versus standard ICUs. For each sensitivity analysis, we repeated the propensity score-matching using the same method as in the main analysis.

All analyses were performed using Stata/MP 16.0 software (StataCorp, College Station, TX, USA). Continuous variables are presented as mean and standard deviation, and categorical variables are presented as number and percentage. All reported *P* values were two-sided, and a *P* value of < 0.05 was considered statistically significant. Because of the potential for type I error due to multiple comparisons, findings for subgroup analyses should be interpreted as exploratory.

## Results

**Fig. 1:**
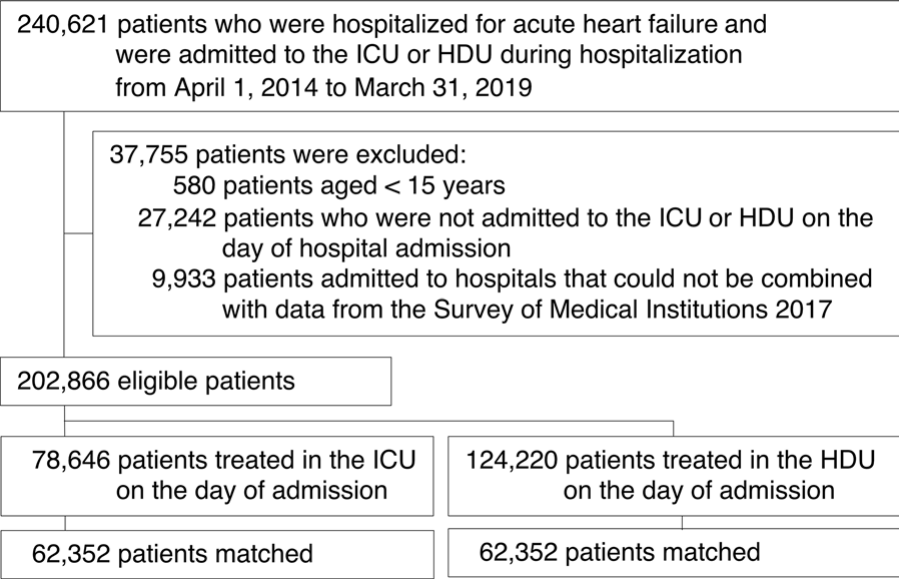
Patient flowchart. ICU, intensive care unit; HDU, high-dependency care unit

In total, 202,866 eligible patients from 737 hospitals with ICU and/or HDU beds were enrolled during the 5-year study period (Fig. 1). Of these patients, 78,646 (39%) were admitted to the ICU and 124,220 (61%) were admitted to the HDU on the day of admission. Of 78,646 patients who were admitted to the ICU on the day of admission, 10,194 (13%) were then transferred to the HDU. Of 124,220 patients who were admitted to the HDU on the day of admission, 4,589 (4%) were then transferred to the ICU.

Table 1 shows the baseline characteristics before and after propensity score matching. In the original cohort, patients in the ICU group tended to be younger; be in a comatose state; use an ambulance; require noninvasive ventilation, intubation, nitrates, nicorandil, dobutamine, noradrenaline, intra-aortic balloon pumping, coronary angiography, percutaneous coronary intervention, and continuous renal replacement therapy; and be admitted to an academic hospital. In contrast, patients in the HDU group tended to have severe dependence, have dementia, be admitted from a nursing home, have atrial flutter/fibrillation, be admitted to a tertiary emergency hospital, and be admitted to a high-volume hospital. One-to-one propensity score matching created 62,352 matched pairs. The distributions of propensity scores before and after the matching are shown in Additional file 1: Figs. S1 and S2. After the propensity score matching, the patients’ characteristics were well balanced between the two groups (Table 1 and Additional file 1: Fig. S3).

Table 2 shows the outcomes before and after the propensity score matching. After the propensity score matching, there was no statistically significant difference in in-hospital mortality between the ICU and HDU groups (10.7% vs. 11.4%; difference, − 0.6%; 95% confidence interval, − 1.5% to 0.2%). Compared with patients in the HDU group, those in the ICU group had significantly longer lengths of hospital stay and higher hospitalization costs. There were no statistically significant differences between the two groups in the length of ICU/HCU stay or complications after admission except stroke.

**Table 2:** Outcomes before and after propensity score matching

Outcomes	Before propensity score matching		After propensity score matching		Difference (95% CI)	*P* value
	ICU	HDU	ICU	HDU		
	(*n* = 78,646)	(*n* = 124,220)	(*n* = 62,352)	(*n* = 62,352)		
In-hospital mortality	8,759 (11.1)	14,672 (11.8)	6,696 (10.7)	7,101 (11.4)	− 0.6 (− 1.5 to 0.2)	0.14
Length of hospital stay, days	26 ± 31	24 ± 24	25 ± 28	24 ± 25	1.1 (0.3 to 1.9)	0.006
Length of ICU/HDU stay, days	5 ± 9	5 ± 6	5 ± 9	5 ± 7	− 0.1 (− 0.4 to 0.2)	0.42
Total hospitalization cost, USD	17,670 (29,246)	12,924 (15,891)	15,934 (23,606)	13,751 (18,347)	2183 (1435 to 2931)	< 0.001
Complications after admission						
Pneumonia	3,199 (4.1)	4,040 (3.3)	2,356 (3.8)	2,155 (3.5)	0.3 (− 0.1 to 0.7)	0.13
Stroke	2,124 (2.7)	2,709 (2.2)	1,631 (2.6)	1,382 (2.2)	0.4 (0.1 to 0.7)	0.003
Endoscopic hemostasis for GI bleeding	417 (0.5)	563 (0.5)	320 (0.5)	313 (0.5)	0.01 (− 0.07 to 0.09)	0.79
Catheter-related bloodstream infection	111 (0.1)	92 (0.1)	79 (0.1)	56 (0.1)	0.04 (0.00 to 0.07)	0.050
*Clostridioides difficile* infection	223 (0.3)	319 (0.3)	183 (0.3)	153 (0.2)	0.05 (− 0.04 to 0.01)	0.28

Data are presented as n (%) or mean ± standard deviation

*ICU* intensive care unit; *HDU* high-dependency care unit; *CI* confidence interval; *USD* United States dollars; *GI* gastrointestinal

The results of the subgroup analyses in the propensity score-matched cohort are shown in Table 3. There was a statistically significant difference in in-hospital mortality between the ICU and HDU groups among patients receiving noninvasive ventilation (9.4% vs. 10.5%; difference, − 1.0%; 95% confidence interval, − 1.9% to − 0.1%) and patients receiving intubation (32.5% vs. 40.6%; difference, − 8.0%; 95% confidence interval, − 14.5% to − 1.5%). There were no statistically significant differences in in-hospital mortality between the two groups in other subgroup analyses.

**Table 3:** Results of subgroup analyses for in-hospital mortality

Analyses	In-hospital mortality, n (%)		Difference, %	*P* value
	ICU	HDU	(95% CI)	
Overall cohort	6,696/62,352 (10.7)	7,101/62,352 (11.4)	− 0.6 (− 1.5 to 0.2)	0.14
Subgroups				
Respiratory support				
No supplemental oxygen	1,087/12,005 (9.1)	1,055/12,106 (8.7)	0.3 (− 0.8 to 1.4)	0.54
Supplemental oxygen	2,393/24,121 (9.9)	2,241/23,535 (9.5)	0.4 (− 0.3 to 1.1)	0.29
Noninvasive ventilation	2,171/23,015 (9.4)	2,442/23,351 (10.5)	− 1.0 (− 1.9 to − 0.1)	0.026
Intubation	1,045/3,211 (32.5)	1,363/3,360 (40.6)	− 8.0 (− 14.5 to − 1.5)	0.015
Intravenous vasodilator				
Yes	2,958/42,058 (7.0)	3,146/41,971 (7.5)	− 0.5 (− 1.0 to 0.1)	0.097
No	3,738/20,294 (18.4)	3,955/20,381 (19.4)	− 1.0 (− 3.1 to 1.1)	0.36
Diuretic				
Yes	3,873/43,123 (9.0)	4,010/43,155 (9.3)	− 0.3 (− 0.9 to 0.3)	0.30
No	2,823/19,229 (14.7)	3,091/19,197 (16.1)	− 1.4 (− 3.6 to 0.8)	0.21
Inotrope				
Yes	1,527/8,735 (17.5)	1,549/8,843 (17.5)	0.0 (− 1.5 to 1.4)	0.96
No	5,169/53,617 (9.6)	5,552/53,509 (10.4)	− 0.7 (− 1.7 to 0.2)	0.13
Vasopressor				
Yes	1,434/5,047 (28.4)	1,522/4,985 (30.5)	− 2.1 (− 4.4 to 0.2)	0.070
No	5,262/57,305 (9.2)	5,579/57,367 (9.7)	− 0.5 (− 1.4 to 0.3)	0.19
Mechanical circulatory support				
Yes	150/583 (25.7)	172/609 (28.2)	− 2.5 (− 7.9 to 2.9)	0.36
No	6,546/61,769 (10.6)	6,929/61,743 (11.2)	− 0.6 (− 1.5 to 0.2)	0.16
Renal replacement therapy				
Yes	306/3,052 (10.0)	282/3,087 (9.1)	0.9 (− 0.8 to 2.6)	0.31
No	6,390/59,300 (10.8)	6,819/59,265 (11.5)	− 0.7 (− 1.6 to 0.1)	0.12

*ICU* intensive care unit; *HDU* high-dependency care unit; *CI* confidence interval

Of the 202,866 patients in the 737 hospitals with ICU and/or HDU beds, 19,512 (10%) were admitted to 157 hospitals with only ICU beds, 23,310 (11%) were admitted to 186 hospitals with only HDU beds, and 160,044 (79%) were admitted to 394 hospitals with both ICU and HDU beds. The results of the sensitivity analyses excluding patients admitted to hospitals with both ICU and HDU beds are shown in Table 4. There were no statistically significant differences between the two groups in in-hospital mortality, length of hospital stay, and complications after admission. Compared with patients in the HDU group, those in the ICU group had significantly shorter lengths of ICU/HCU stay and higher hospitalization costs.

**Table 4:** Results of sensitivity analyses excluding patients admitted to hospitals with both ICU and HDU beds after propensity score matching

Outcomes	After propensity score matching			
	ICU	HDU	Difference	*P* value
	(*n* = 11,527)	(*n* = 11,527)	(95% CI)	
In-hospital mortality	1,465 (12.7)	1,480 (12.8)	− 0.1 (− 1.9 to 1.6)	0.88
Length of hospital stay, days	27 ± 26	28 ± 27	− 0.9 (− 2.9 to 1.0)	0.33
Length of ICU/HDU stay, days	5 ± 7	5 ± 7	− 0.9 (− 1.4 to − 0.5)	< 0.001
Total hospitalization cost, USD	14,501 (17,070)	12,584 (11,462)	1916 (918 to 2914)	< 0.001
Complications after admission				
Pneumonia	431 (3.7)	496 (4.3)	− 0.6 (− 1.4 to 0.3)	0.18
Stroke	293 (2.5)	253 (2.2)	0.3 (− 0.2 to 0.9)	0.24
Endoscopic hemostasis for GI bleeding	53 (0.5)	59 (0.5)	− 0.05 (− 0.24 to 0.13)	0.58
Catheter-related bloodstream infection	21 (0.2)	13 (0.1)	0.07 (− 0.03 to 0.17)	0.18
*Clostridioides difficile* infection	46 (0.4)	47 (0.4)	0.00 (− 0.25 to 0.23)	0.94

Data are presented as n (%) or mean ± standard deviation

*ICU* intensive care unit; *HDU* high-dependency care unit; *CI* confidence interval; *USD* United States dollars; *GI* gastrointestinal

Of 78,646 patients who were admitted to the ICU on the day of admission, 9,747 (12%) were admitted to resource-rich ICUs and 68,899 (88%) were admitted to standard ICUs. The results of the sensitivity analyses between patients in resource-rich ICUs versus HDUs, standard ICUs versus HDUs, and resource-rich ICUs versus standard ICUs were also similar to those of the main analyses (Table 5).

**Table 5:** Results of sensitivity analyses of in-hospital mortality between patients in resource-rich ICUs versus HDUs, standard ICUs versus HDUs, and resource-rich ICUs versus standard ICUs

Sensitivity analyses	After propensity score matching		
	In-hospital	Difference, %	*P* value
	Mortality, *n* (%)	(95% CI)	
Resource-rich ICU vs. HDU			
Resource-rich ICU	956/9661 (9.9)	− 1.1 (− 2.4 to 0.4)	0.16
HDU	1065/9661 (11.0)		
Standard ICU vs. HDU			
Standard ICU	6272/57,384 (10.9)	− 0.6 (− 1.5 to 0.3)	0.22
HDU	6596/57,384 (11.5)		
Resource-rich ICU vs. standard ICU			
Resource-rich ICU	986/9745 (10.1)	− 1.2 (− 2.6 to 0.2)	0.085
Standard ICU	1106/9745 (11.3)		

*ICU* intensive care unit; *HDU* high-dependency care unit; *CI* confidence interval

## Discussion

In this nationwide cohort study of patients with acute heart failure, there was no significant difference in in-hospital mortality between the ICU and HDU groups in the entire cohort. This finding was consistent in the sensitivity analyses comparing structure and staffing models with different intensivist staffing models and nurse-to-patient ratios. Meanwhile, the ICU group had significantly lower in-hospital mortality than the HDU group among patients receiving noninvasive ventilation and intubation.

Unlike previous studies on general ICUs [25], one previous study on CICUs [13], and recommendations from academic societies [4, 8–10], the present study showed that the different structure and staffing models were not associated with reduced mortality in patients with acute heart failure. One possible reason is that intensivist staffing failed to produce a benefit over specialized care by cardiologists for patients with acute heart failure. Intensivists have the potential to improve patient care and outcomes through their specialist knowledge of organ support therapies, extensive experience with critically ill patients, and higher compliance with evidence-based protocols [26]. However, most of the patients in the present cohort did not require organ support therapies including invasive mechanical ventilation, mechanical circulatory support, and renal replacement therapy. Therefore, the specialty of intensivists might not be utilized.

Another possible reason is that a nurse-to-patient ratio of 1:4 or 1:5 was not inferior to a ratio of 1:2 in the present cohort of patients with acute heart failure. Previous studies have shown that inadequate nurse staffing is associated with increased mortality and that critically ill patients demand high nurse workloads [27, 28]. However, because most of the patients in the present cohort only required single organ support for respiratory failure or circulatory failure without invasive treatments, a nurse-to-patient ratio of 1:4 may be adequate for care of such patients requiring a lower workload.

The above reasons may also explain the lower mortality in the ICU group in the subgroup of patients receiving noninvasive ventilation and intubation. The recent ICU admission guideline recommends that patients with invasive treatments such as mechanical ventilation have the highest priority for ICU admission [7]. Therefore, this study may support ICU triage based on the combination of patient type and invasive interventions rather than based on the diagnosis alone.

The findings of this study should be interpreted carefully. Because this was not a clinical trial, no causation can be inferred. The findings of this study do not support the treatment of all patients with acute heart failure in the HDU instead of the ICU. In critically ill patients, overtriage is recommended and preferable to undertriage [7]. Therefore, the present study shows one possibility that care in the HDU for patients with non-advanced acute heart failure may be cost-effective without compromising quality. Further studies are needed to verify our findings and to examine how other structure and staffing models of CICUs impact patient outcomes.

The present study has some limitations. First, we used a multicenter, real-world database in Japan, and there was no standard protocol for critical care admission. Therefore, admission to the HDU rather than the ICU for patients with acute heart failure was not random and was based on the decision of the attending physicians or circumstances of each hospital, which may have led to confounding by indication. We attempted to control for measured confounders in the propensity score analyses; however, there still may have been unmeasured confounders such as vital signs [29], prior hospitalization for heart failure [30] and ejection fraction [31, 32]. Therefore, we conducted a sensitivity analysis excluding patients admitted to hospitals with both ICU and HDU beds and confirmed that the impact of this bias would be small. Second, because the severity of illness and invasive interventions might modify the effect of ICU admission on in-hospital mortality, the average effect will differ between different populations. Furthermore, the definitions of ICU and HDU are not consistent among countries. Therefore, the results of this study may not be generalizable to other populations of patients who receive care in the CICU. Third, there are other unmeasured factors that affect assessment of organizational structure and staffing models, such as closed or open ICU models [5], the presence of cardiac intensivists, general ICUs or cardiac-specialized ICUs, the number of full-time or non-full-time doctors, and physician’s specialty (cardiologist or intensivist). Therefore, future studies should include these variables to clarify which organizational structure and staffing models are most effective in reducing mortality.

## Conclusion

The present study showed that care in the ICU was not associated with lower in-hospital mortality than care in the HDU among the entire cohort with acute heart failure. However, critical care in ICUs was associated with lower in-hospital mortality than critical care in HDUs among patients receiving noninvasive ventilation and intubation.

## Supplementary Information

**Additional file 1: Table S1.** Japanese medical procedure codes used to define ICUs and HDUs. **Fig. S1.** Distributions of propensity scores before propensity score matching in the main analysis. **Fig. S2.** Distributions of propensity scores after propensity score matching in the main analysis. **Fig. S3.** Balance of the covariates before and after propensity score matching in the main analysis

## Data Availability

The dataset analyzed in the current study is not publicly available because of contracts with the hospitals providing data to the database.

## Abbreviations

CICU: Cardiac intensive care unit
ICU: Intensive care unit
HDU: High-dependency care unit
ICD-10: International Classification of Diseases, Tenth Revision
