# Supplementary material for: Intensive care unit versus high-dependency care unit for patients with acute heart failure: a nationwide propensity score-matched cohort study
Source: J Intensive Care. 2021 Dec 20;9:78. doi: 10.1186/s40560-021-00592-2 (PMC8686245; doi:10.1186/s40560-021-00592-2)
Supplement: Supplementary file 1 — Additional file 1: Table S1. Japanese medical procedure codes used to define ICUs and HDUs. Fig. S1. Distributions of propensity scores before propensity score matching in the main analysis. Fig. S2. Distributions of propensity scores after propensity score matching in the main analysis. Fig. S3. Balance of the covariates before and after propensity score matching in the main analysis [file 40560_2021_592_MOESM1_ESM.docx]

*Supplementary Material*

**Intensive care unit versus high-dependency care unit for patients with acute heart failure: a nationwide propensity score-matched cohort study**

Hiroyuki Ohbe, MD, Hiroki Matsui, MPH, Hideo Yasunaga, PhD

Department of Clinical Epidemiology and Health Economics, School of Public Health, The University of Tokyo, 7-3-1 Hongo, Bunkyo-ku, Tokyo 1130033, Japan

**List of Supplemental Tables and Figures**

**Supplemental Tables:**

**Supplemental Table 1.** Japanese medical procedure codes used to define ICUs and HDUs

**Supplemental Figures:**

**Supplemental Figure 1.** Distributions of propensity scores before propensity score matching in the main analysis

**Supplemental Figure 2.** Distributions of propensity scores after propensity score matching in the main analysis

**Supplemental Figure 3.** Balance of the covariates before and after propensity score matching in the main analysis

**Supplemental Table 1.** Japanese medical procedure codes used to define ICUs and HCUs

|  |  |  | Cost per day, | Intensivist | Nurse-to-patient |
| --- | --- | --- | --- | --- | --- |
| Name | Code | Description | USD* | staffing | ratio |
| Resource-rich ICU | A3011 | ICU management fee 1 | 1292 | ≥ 2 | 1:2 |
| Resource-rich ICU | A3012 | ICU management fee 2 | 1292 | ≥ 2 | 1:2 |
| Standard ICU | A3002 | Emergency and critical care unit management fee 2 | 1072 | – | 1:2 |
| Standard ICU | A3004 | Emergency and critical care unit management fee 4 | 1072 | – | 1:2 |
| Standard ICU | A3013 | ICU management fee 3 | 882 | – | 1:2 |
| Standard ICU | A3014 | ICU management fee 4 | 882 | – | 1:2 |
| HDU | A3001 | Emergency and critical care unit management fee 1 | 929 | – | 1:4 |
| HDU | A3003 | Emergency and critical care unit management fee 3 | 929 | – | 1:4 |
| HDU | A301-21 | HDU management fee 1 | 623 | – | 1:4 |
| HDU | A301-22 | HDU management fee 2 | 386 | – | 1:5 |

The definition of resource-rich ICU was a separate unit providing critical care services with at least one physician on site 24 hours per day, at least two intensivists working full-time, around-the-clock nursing, the equipment necessary to care for critically ill patients, a nurse-to-patient ratio of 1:2, ≥ 20 m2 per ICU bed, and a medical engineer in the hospital 24 hours per day. The definition of standard ICU was a separate unit providing critical care services with at least one physician on site 24 hours per day, around-the-clock nursing, the equipment necessary to care for critically ill patients, and a nurse-to-patient ratio of 1:2. The definition of standard ICU was separate unit providing intermediate critical care services with around-the-clock nursing, the equipment necessary to care for critically ill patients, and a nurse-to-patient ratio of 1:4 or 1:5.

*Cost per day is shown for the first 7 days after ICU/HDU admission.

ICU, intensive care unit; HDU, high-dependency care unit; USD, United States dollars

**Supplemental Figure 1.** Distributions of propensity scores before propensity score matching in the main analysis


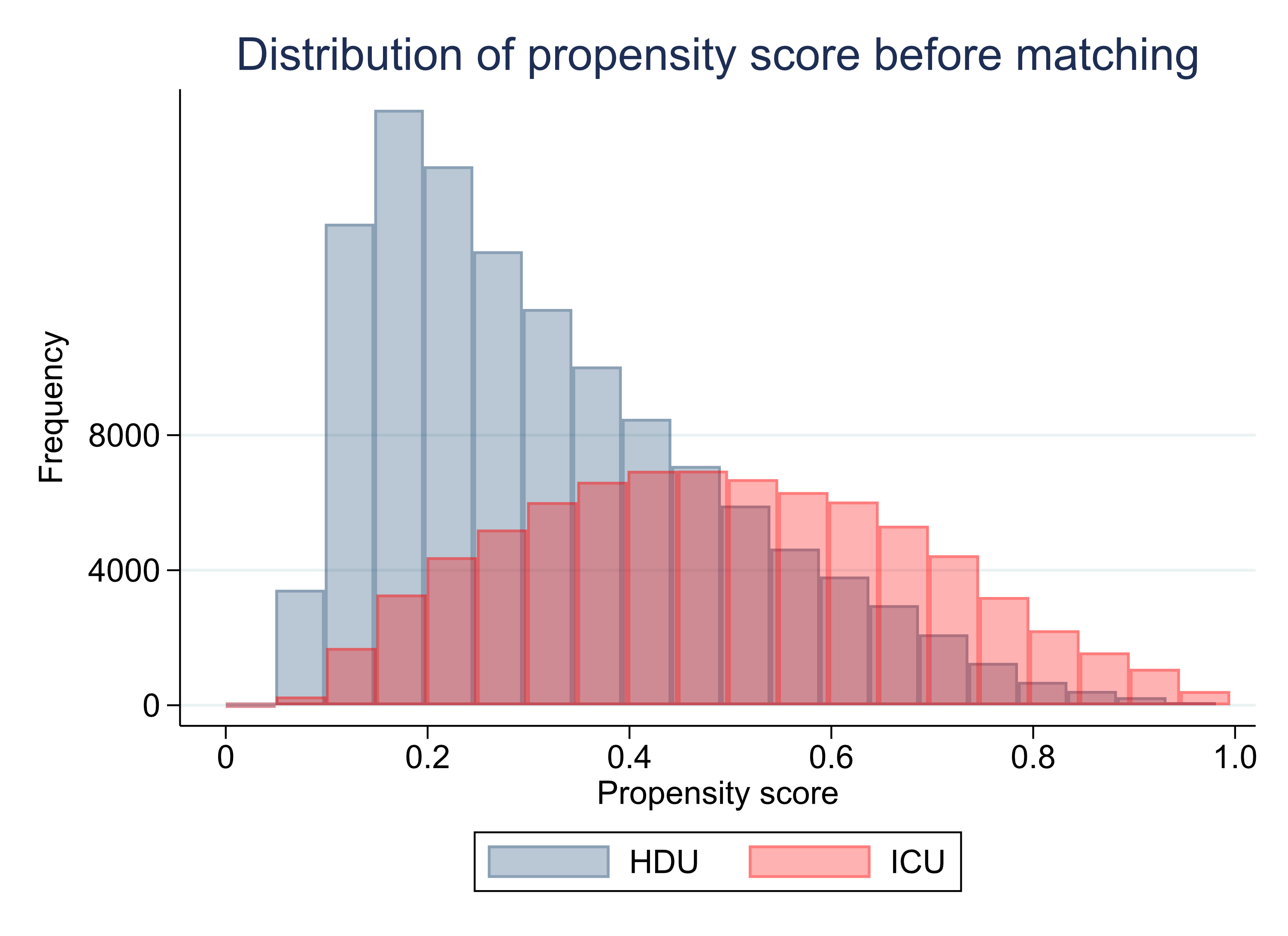


ICU, intensive care unit; HDU, high-dependency care unit

**Supplemental Figure 2.** Distributions of propensity scores after propensity score matching in the main analysis


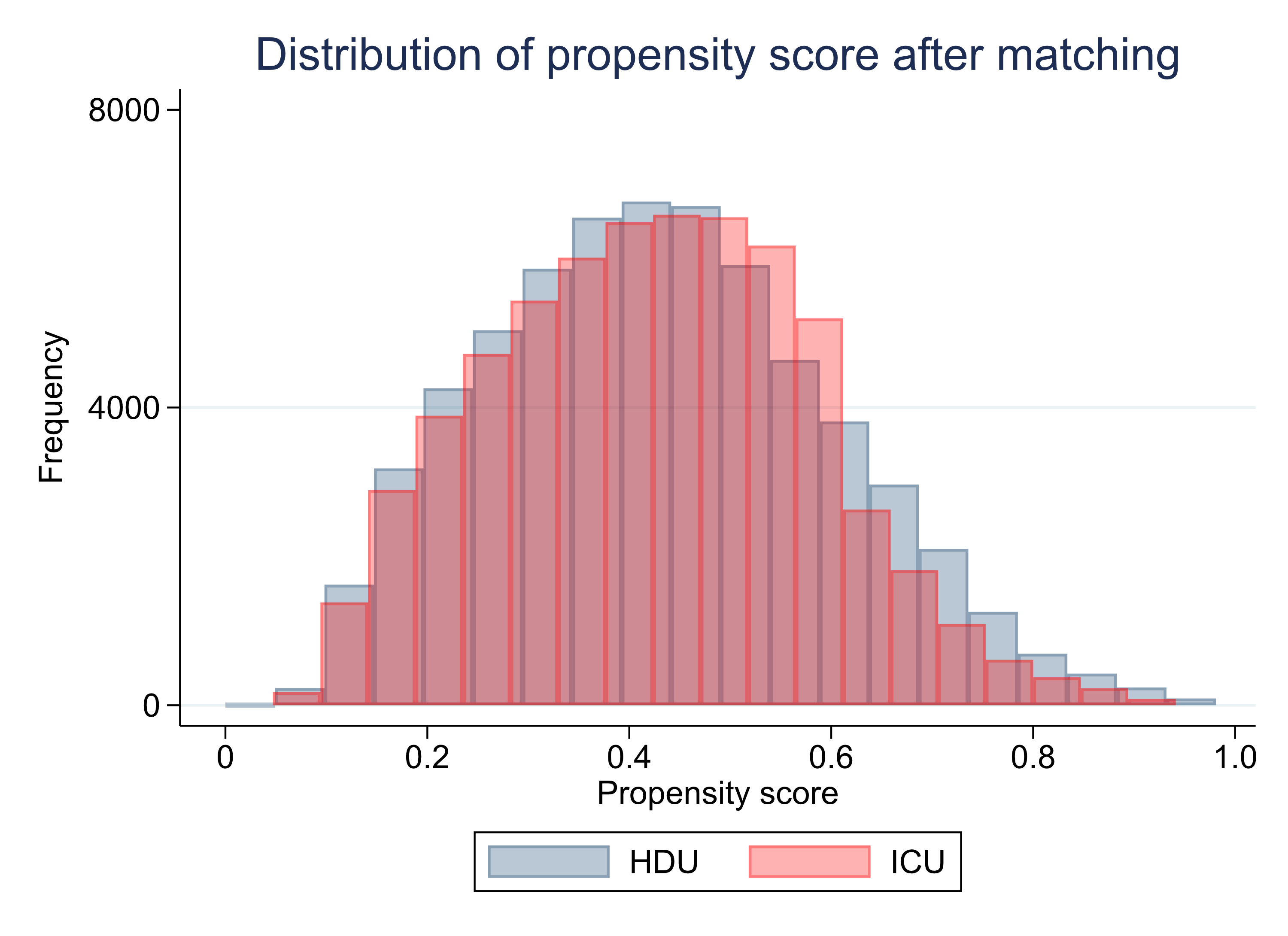


ICU, intensive care unit; HDU, high-dependency care unit

**Supplemental Figure 3.** Balance of the covariates before and after propensity score matching in the main analysis


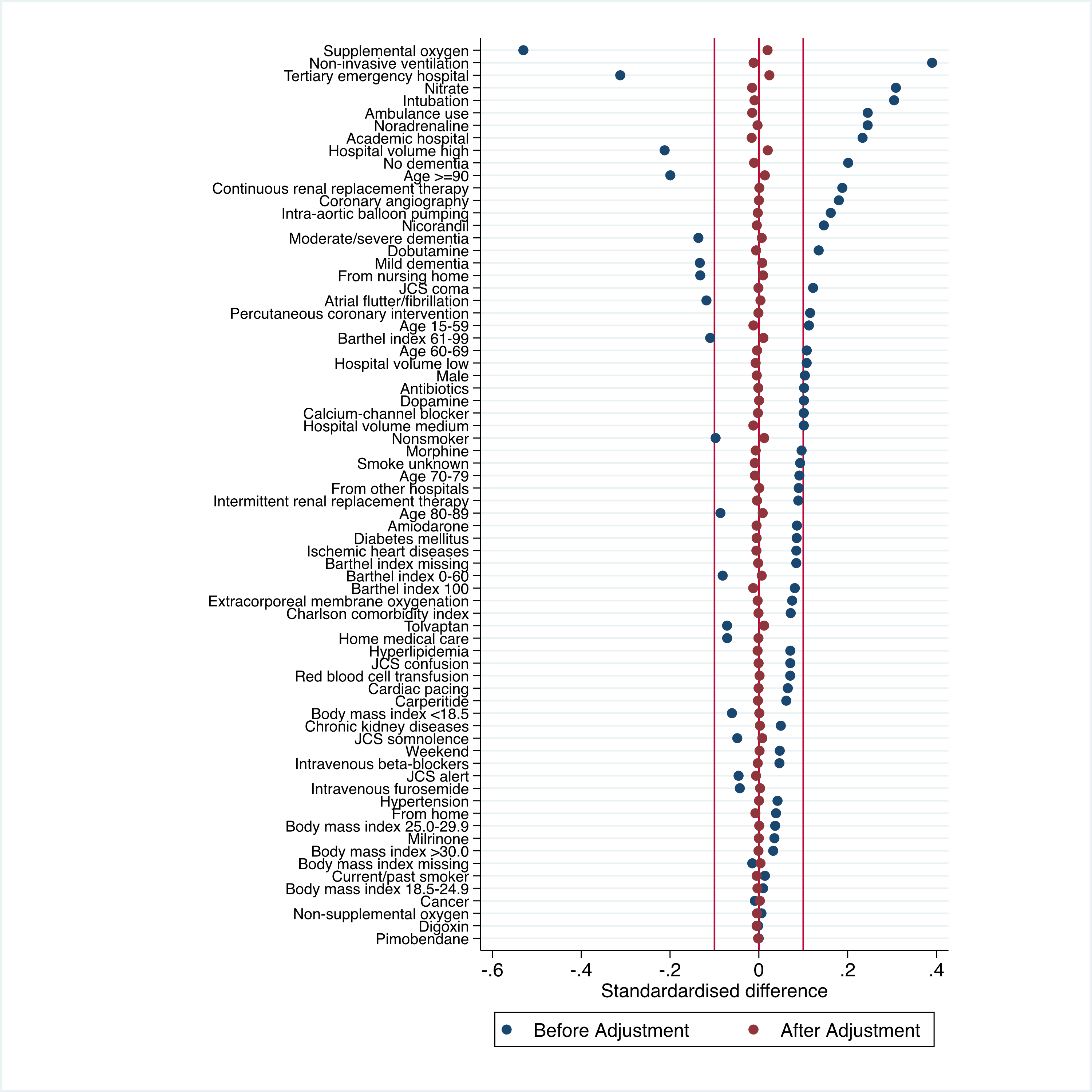


JCS, Japan Coma Scale
